# A systematic review of high quality randomized controlled trials investigating motor skill programmes for children with developmental coordination disorder

**DOI:** 10.1177/0269215516661014

**Published:** 2016-08-01

**Authors:** Nick Preston, Sara Magallón, Liam JB Hill, Elizabeth Andrews, Sara M Ahern, Mark Mon-Williams

**Affiliations:** 1 School of Psychology, University of Leeds, Leeds, UK; 2 Faculty of Education and Psychology, University of Navarre, Pamplona, Spain; 3 Bradford Institute for Health Research, Bradford, UK

**Keywords:** Developmental coordination disorder, motor impairment, motor skill programmes, randomized control trial, systematic review

## Abstract

**Objective:**

To identify effective motor training interventions for children with developmental coordination disorder from research graded as high quality (using objective criteria) for the purpose of informing evidence-based clinical practice.

**Data sources:**

We followed the guidance for conducting systematic reviews issued by the Centre for Reviews and Dissemination. Six OvidSP electronic databases (AMED, All EBM reviews (including Cochrane), Embase, Ovid MEDLINE, PsychARTICLES Full Text, PsycINFO) were searched systematically. We aimed to retain only randomized control trials and systematic reviews of randomized control trials, defined as the highest level of evidence by the Oxford Centre for Evidence-Based Medicine. We searched reference lists of retained articles to identify further appropriate articles.

**Review methods:**

Two reviewers critically appraised and categorized articles by effect size (including confidence intervals), inclusion of power calculations and quality using the Physiotherapy Evidence Database (PEDro) scale. Only studies scoring seven or more on the PEDro scale (classed by the PEDro as high reliability) were retained.

**Results:**

No systematic reviews met our criteria for inclusion from 846 articles yielded by the systematic search. Nine randomized control trials investigating 15 interventions to improve motor skills met our inclusion criteria for ‘high quality’. Nevertheless, not all included studies were adequately powered for determining an effect.

**Conclusion:**

Large effect sizes associated with 95 % confidence intervals suggest that ‘Neuromotor Task Training’, ‘Task-oriented Motor Training’ and ‘Motor Imagery + Task Practice Training’ are the most effective reported interventions for improving motor skills in children with developmental coordination disorder.

## Introduction

Developmental Coordination Disorder describes deficits in the acquisition and automation of motor procedures^1^ that have deleterious impacts on a child’s life, including: Lower levels of academic attainment,^1–3^ reduced participation in social and leisure activities^2,3^ and increased risk of further health problems^4–6^ (both physical and mental). In the general population, developmental coordination disorder is estimated to affect 5%–6% of children.^2^ Parents of children with developmental coordination disorder frequently express frustration at a lack of appropriate support^7,8^ and report dissatisfaction with the quality of the therapy services offered to their children.^9^ Such complaints imply that some of the therapeutic approaches being used at present may not be optimal and that the evidence base supporting their usage may require more rigorous evaluation.

Recently, guidelines^10^ for the treatment of children with developmental coordination disorder were informed by a systematic review^11^ that studied the evidence base (published between 1995 and 2011) underpinning the wide variety of interventions used to support children with developmental coordination disorder. It included evaluations of occupational and physical therapy, pharmacological, dietary and education-based interventions,^10–14^ and concluded that in diagnosed cases of developmental coordination disorder, intervention was generally better than no intervention. However, while the review^11^ was particularly thorough in terms of the breadth of literature it encompassed, its authors noted that the majority of studies selected (17 out of 24) relied on insufficiently robust research designs (see Oxford Centre for Evidence-based Medicine definitions^15^) and the results were therefore potentially attributable to uncontrolled confounding factors. Moreover, they assessed three of the experimental studies they reviewed as ‘weak’ and only 11 as ‘moderate’ on the PEDro (quality of evidence) Scale.^11,16^ This meant that while a cumulative total of 912 children were identified as having participated in the 24 experimental studies reviewed, less than a third took part in studies that Smits-Engelsman et al.^11^ rated as being of ‘high’ methodological quality (i.e. a PEDro score ⩾7^16^ and Oxford Level of Evidence: Ib).^15^

This raises the question of whether the evidence base for treating developmental coordination disorder should be re-examined with greater stringency. The primary purpose of a systematic review in healthcare policy making is to provide a summary of all the ‘best available research evidence’,^17^ as opposed to all available research evidence regardless of quality. The current systematic review aims to provide such a summary by identifying motor training interventions for improving movement skills of children with developmental coordination disorder from systematic reviews and randomized controlled trials assessed as being of high quality (using objective standardized criteria) for the purpose of informing evidence-based clinical practice.

## Method

For systematic reviews that evaluate the effects of health interventions, both the National Institute for Health Research Health Technology Assessment programme and the National Institute for Health and Care Excellence recommend the guidelines published by the Centre for Reviews and Dissemination.^17^ These guidelines suggest that reporting of systematic reviews should use the PRISMA^18^ flowchart and checklist. The conduct and reporting of this systematic review therefore adopts the guideline principles published by the Centre for Reviews and Dissemination^17^ and the PRISMA Statement,^18^ respectively.

### Identification of studies

Systematic literature searches^17^ using the terms and strategy shown in Table S1 (supplementary material, available online) were conducted in six OvidSP electronic databases: AMED, All EBM reviews (this includes Cochrane), Embase, Ovid MEDLINE, PsychARTICLES Full Text and PsycINFO. Supplementary to this, a hand search of the reference lists of the articles selected for full-text review and recent editions (previous six months) of the journals in which these articles were published was also carried out.

### Inclusion and exclusion criteria

Because two other reviews^11,19^ identified that there were no high quality randomized controlled studies or other relevant systematic reviews before 2000, we filtered articles identified by the electronic literature searches to include only articles published between the year 2000 and the date of the search (1 March 2016). Each title and abstract was then studied independently by two reviewers and articles short-listed for full-text review if this information suggested the article was potentially either a randomized controlled trial or a systematic review of randomized controlled trials investigating motor interventions designed to improve movement skills of children with developmental coordination disorder aged six to 12 years old. Motor interventions were defined as those interventions that involved a physical exercise programme such as sports, exercise, movement, balance and motor training activities. Lack of agreement between reviewers on this or any other evaluative points was settled through discussion, with such discussions always involving consultation with at least one further reviewer.

### Quality assessment

Full-text reviewing of short-listed articles followed, with articles being rated in terms of their Level of Evidence (according to the Oxford Centre for Evidence Based Medicine^15^) and for quality using the PEDro scale^16^ (shown in Table 1, available online), if they were a randomized controlled trial. Only those articles that reviewers agreed were classifiable as ‘high’ in terms of their level of evidence were retained. ‘High’ was defined as: A systematic review of randomized controlled trials (Oxford Level Ia)^15^ or a randomized controlled trial (Oxford Level Ib)^15^ and, in the case of randomized controlled trials, also scoring seven out of 11 or higher on the PEDro scale (i.e. classed as high reliability).^11,16^

### Data extraction and synthesis of results

Data were extracted from studies into a Microsoft Excel spreadsheet with headings based on the guidelines from the Centre for Reviews and Dissemination.^17^ From this information, effect sizes and 95% confidence intervals were calculated to better evaluate the outcomes of studies.^20,21^

## Results

### Study selection

**Figure 1.**
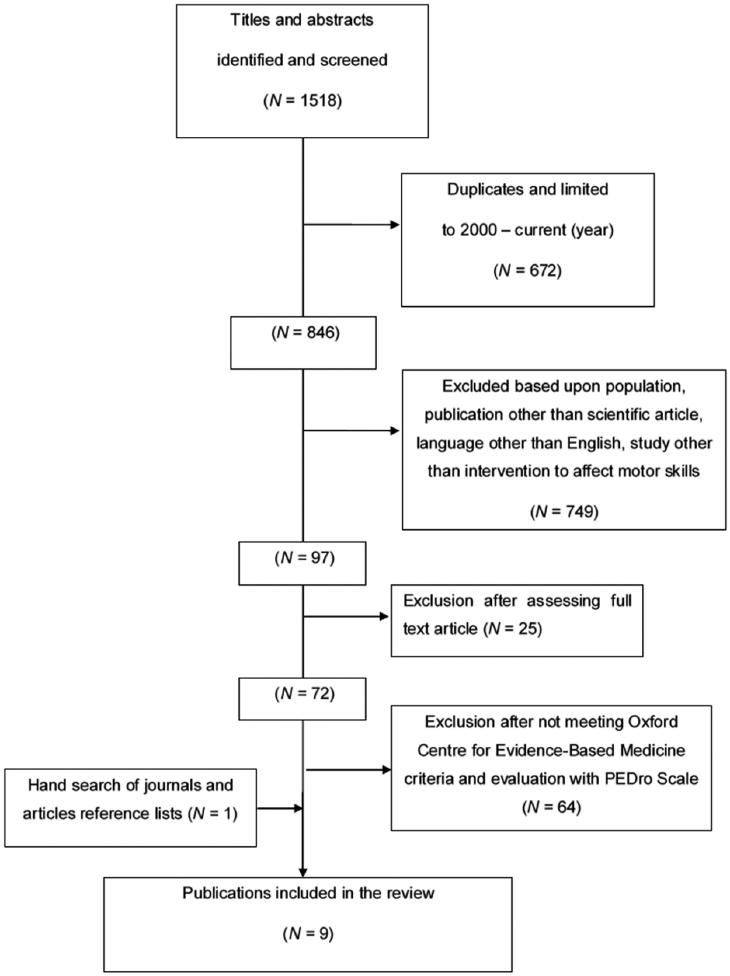
Flowchart of the study selection process.

The article selection process summarized in Figure 1 produced a final total of nine randomized controlled trials included in this review, including one added after hand search of journals. No systematic reviews of randomized controlled trials were identified. Meanwhile at the full-text review stage (*n* = 72), in which 64 articles^4,10–12,22–81^ were rejected for not meeting our inclusion criteria, Cognitive Orientation to Occupational Performance (16 studies) and Neuromotor Task Training (four studies) were the most widely represented ‘task-orientated’ type interventions investigated.

We included only three of the 26 articles in the review by Smits-Englesman et al.^11^ Of the 23 articles not included, four articles did not evaluate motor skills interventions,^10,12–14^ 15 did not meet our evidence-based criteria (Oxford Centre for Evidence-based Medicine criteria^15^ and PEDro scores^16^),^19,33,35,36,39,46,52,58,66,69,70,73,74,77,82^ two were rejected because they did not meet our search criteria^31,32^ and two were dated before 2000.^29,30^

Five of the 15 randomized controlled trials that we rejected were scored by Smits-Engelsman et al. as ⩾7 on the PEDro scale, but were omitted from this review when we scored them as <7. PEDro scores for these rejected studies were identical between our two scorers, and this was reinforced when each study was re-reviewed by the scorers again after noting the difference with the earlier review. Reasons for low PEDro scores were generally similar between the studies. Namely: Participants were not randomly selected;^82^ single case study methodology;^52^ participants were not randomly allocated to groups;^70^ allocation to groups was not concealed; groups were not similar at baseline regarding the most important prognostic indicators; no attempts to blind participants (neither therapists who administered the therapy nor outcome assessors);^33,39^ not all children for whom outcome measures were available received the intervention or control condition as allocated and measures of central tendency and variability for at least one key outcome were not provided.^39^

### Study characteristics

A total of 311 children (median sample size 28.5, range 13 to 58) with developmental coordination disorder participated in the nine randomized controlled trials, which were characterized by focusing mainly on three different therapeutic approaches: Sports, task-oriented and process-oriented. Descriptive statistics and an outline of each of the nine final studies included in the review are given in Table S2 (supplementary material). Seven studies were carried out in schools, two in hospitals, one in a university and one in a swimming pool. Within the nine randomized controlled trials included in the review, seven used only the Movement Assessment Battery for Children, 2nd edition.^83^ The Movement Assessment Battery for Children-2^83^ superseded the original 1992 version of the Movement Assessment Battery for Children^84^ in 2007 and covers the areas of manual dexterity, throwing and catching, and balance. One study used the Bruininks-Oseretsky Test of Motor Proficiency,^85^ a test of fine and gross motor abilities. The Movement Assessment Battery for Children-2 and Bruininks-Oseretsky Test of Motor Proficiency are the only assessment tools recommended by name in the European Academy of Childhood Disability guidelines for diagnosing developmental coordination disorder.^86^ One study used the somato-sensory visual, vestibular ratio (evaluated using a sensory organization test with a ‘dynamic posturography’ machine), and a measure of unilateral stance centre of pressure sway velocity (which was used to generate an ‘equilibrium score’).^87^ Programmes ranged from five to 12 weeks, one to five times per week with each session taking between 30 and 60 minutes.

### Level and quality of evidence

Out of 11 points (the maximum score on the PEDro scale), three of the nine randomized controlled trials scored nine, four randomized controlled trials scored eight and two scored seven points. All randomized controlled trials specified the eligibility criteria, analysed data by ‘intention to treat’, reported between-group statistical comparisons for the key outcomes and randomly allocated subjects to groups. In four randomized controlled trials the allocation was not concealed, and in two studies the randomization procedure was not clear, with allocation influenced by parental consent. In one randomized controlled trial the allocated groups were not similar at baseline regarding the most important prognostic indicators. One randomized controlled trial measured outcomes in only 76.2% of allocated participants.

None of the nine randomized controlled trials included blinded participants and most interventions were conducted by professionals that were unblinded to the children’s group allocation. In many cases this is unavoidable owing to the nature of physical rehabilitation studies. However, some studies evaluated outcomes using unblinded assessors. Six studies included power calculations,^87–92^ two of them post-hoc calculations,^88,91^ but only two of the six achieved adequate power.^87,89^

### Results of individual studies, synthesis of results and additional analyses

**Figure 2.**
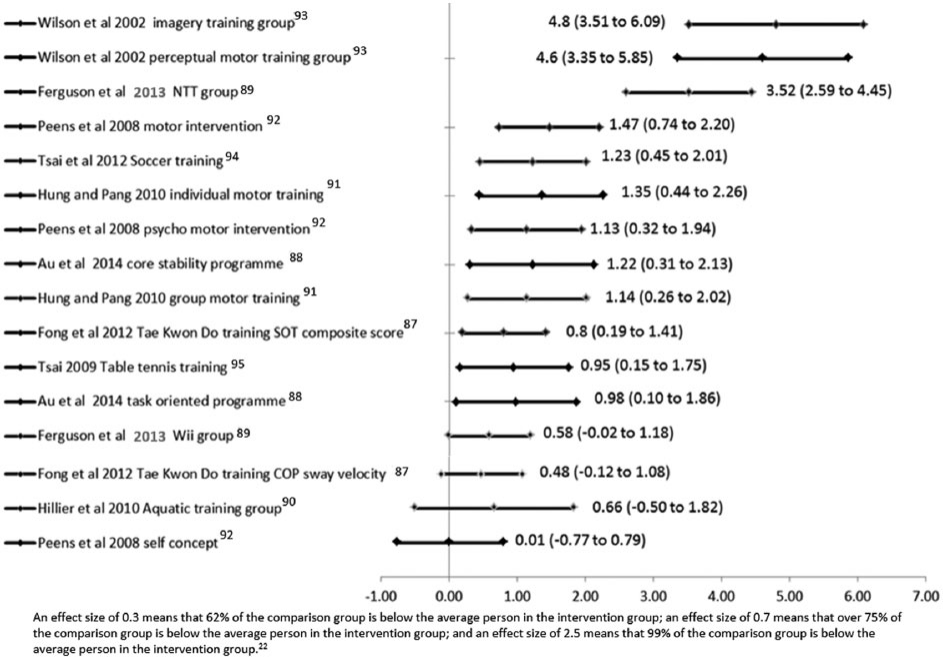
Effect sizes and associated 95% confidence intervals for each intervention in every study.^20^

Because of the small number of studies identified and the differences between them, it was not appropriate to perform a meta-analysis. Each study’s outcome data are given in Table S3 (supplementary material), but for comparison between interventions, effect sizes and 95% confidence intervals are presented in Figure 2, which show either unfavourable or favourable results against or in support respectively of each intervention.

**Figure 3.**
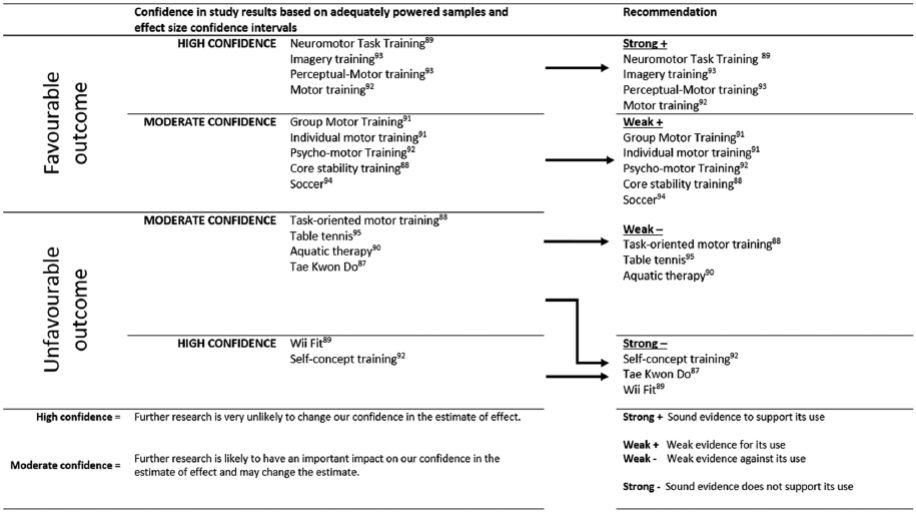
Confidence in results and strength of evidence for each intervention.

Figure 3 summarizes the results of the evidence synthesis based on whether: The study was adequately powered and on the effect size confidence intervals; the evidence supporting the intervention (favourable results) or against the intervention (unfavourable results) is strong or weak.

## Discussion

The aim of this systematic review was to identify the highest quality evidence regarding the use of motor training interventions in children with developmental coordination disorder. To achieve this aim, we used more restrictive inclusion criteria (Oxford Evidence levels Ia or Ib and, in the latter case, a PEDro score^16^ of seven or higher for randomized controlled trials) than previous systematic reviews.^11,69^ This was a principled decision that ensures this systematic review can make recommendations about the likely effectiveness and ineffectiveness of the interventions under investigation with a higher degree of confidence than possible in previous reviews of this literature. Thus this review provides a more rigorous test of the evidence base for intervention to better inform public policy making.

No systematic reviews met our criteria and it transpired that very few of the trials included in Smits-Engelsman et al.^11^ also feature in the current review, primarily owing to the more stringent inclusion criteria concerning methodological quality. This difference is also likely influenced by contextual factors around the Smits-Engelsman et al.’s review,^11^ which was required to encompass the evidence base (irrespective of quality) for all interventions at that time (2012) being used with children with developmental coordination disorder – so that these interventions could all receive appraisal while formulating the European Academy of Childhood Disability’s guidelines on intervention in developmental coordination disorder.^10^ As a result, this is the first relevant review to only consider evidence from studies defined as randomized controlled trials in terms of the Oxford Levels of Evidence^15^ (i.e. all level Ib studies).

Our review broadly agrees with the conclusions of earlier reviews,^11,69^ but substantively contrasts with Smits-Engelsman et al.^11^ in not including any randomized controlled trials investigating Cognitive Orientation to Occupational Performance^39^ (a form of task-orientated intervention). None of the studies investigating this approach were rated as being sufficiently methodologically robust to allow reliable evaluation of efficacy, despite this being one of the most commonly assessed approaches. This is an intervention method that Smits-Engelsman et al.^11^ conclude ‘should be prescribed with some confidence to children with Developmental Coordination Disorder’. However, based on the current review’s evidence, we would suggest that methodologically robust research is necessary before such a strong recommendation can be made.

To aid the development of recommendations for and against the use of specific interventions we evaluated the results based on the inclusion of power calculations, whether studies achieved adequate power and calculated effect sizes along with their confidence intervals. The resultant categories are discussed as follows.

### Effective interventions (strong evidence)

Two adequately powered studies demonstrated very large improvements for three interventions^89,93^ including the Neuromotor Task Training approach,^89^ which is also endorsed in Smits-Engelsman et al.’s^11^ review. With respect to Neuromotor Task Training, children (*n* = 27) practised components of soccer, netball, variations of tagging games and other popular games in workstations under the guidance of therapists who manipulated aspects of the environment and tasks as needed. The comparison group was a Wii Fit training programme group (*n* = 19). After nine weeks of training (two 45–60-minute sessions per week) the Neuromotor Task Training group showed a very large, statistically significant improvement in Movement Assessment Battery for Children-2 scores, as illustrated in Figure 2. This was well above the score difference said to be essential for achieving improvement in motor skills of children with developmental coordination disorder.^87^ Notably, the manual dexterity subscores also showed a statistically significant and very large improvement.

Wilson et al.^93^ also produced evidence that potentially supports practicing motor skills as a means of producing large improvements in children’s movement skills. They found two particular interventions to be effective. One focused on motor imagery training of motor tasks and the other entailed more active perceptual-motor training, involving fine and gross motor learning and perceptual-motor activities that included balance and ball skills tasks. This latter intervention was similar in makeup to another that was independently found to produce moderately positive effects in a different, adequately powered, sample.^92^

All three of these motor intervention programmes^89,92,93^ had features in common: A task-oriented approach was a key feature and, although two were group based, they were tailored to the individual needs and particular interests of the children. The use of equipment, like hoops, ropes and ladders, and outdoor games, were also a core feature.

### Potentially effective interventions (weak +)

While Peens et al.^92^ presented some evidence that their motor skills programmes were an effective intervention, their comparison group of a psycho-motor intervention programme was much less effective. However, the psycho-motor group was underpowered and the control group also showed a medium improvement, which may suggest that the results of this study are not representative of true effects. A study by Hung and Pang^91^ also detected a small positive change in scores in two groups undergoing motor skills training in groups (*n* = 12) or individual-based training (*n* = 11), but these very small groups mean that the study is unlikely to be able to detect a true effect.

Tsai et al.^94^ carried out an intensive ten-week (five 50-minute sessions per week) soccer training programme in school-based groups of 9–10-year-old children; these children were ‘quasi-randomized’ to intervention (*n* = 16) or non-intervention control groups (*n* = 14). This study indicated a moderate change in motor skills. One possible reason for a smaller change than less intensive programmes is that soccer training is likely to impact mainly on mobility and balance, and much less so on upper limb and fine motor movements. The lack of power calculations and lack of true randomization also limit the quality of this study.

Au et al.^88^ carried out an underpowered study of a process-oriented approach through a ‘Core Stability’ programme. The results suggested that process-oriented training could have a small effect on motor skills, but the power of the study to detect these effects limits its conclusions. It is worth noting that 80% of parents in this study showed a preference for group-based training over individual-based intervention.

### Potentially ineffective interventions (weak –)

The underpowered study by Au et al.^88^ also investigated task-oriented motor skills training and reported a negligible effect on motor skills. Of potential importance, there was a strong correlation between children’s participation in a home exercise programme and improvement in outcome scores, for example correlation with motor scores.

Hillier et al.^90^ suggested that aquatic therapy improves motor skills of children with developmental coordination disorder and recommended that aquatic therapy is used by therapists but they recruited only a third of their calculated sample size. They report results for a promising effect on motor skills, but when between-participant variability is fully taken into account (see Figure 2), the results suggest benefits are unlikely to be reliably positive in the wider population. Similarly, Tsai^95^ concluded that children undertaking an intensive 10-week table tennis training programme showed a significant improvement in motor skills, but our calculations imply that the effect is negligible in terms of its clinical significance.

### Ineffective interventions (strong evidence)

One well-designed, high-quality powered randomized controlled trial^89^ concluded that Wii Fit training produced moderate improvements in motor skills, but our calculations (see Figure 2) suggest that Wii training had no effect.

Fong et al.^87^ compared children participating in an intensive Tae Kwon Do training group to a control group (undergoing no Tae Kwon Do training). At best, there were negligible gains in children’s balance, but the authors did not report whether this translated to functional improvements.

Peens et al.^92^ tested a psychological intervention programme against two other programmes (described above) to evaluate whether it improved motor difficulties and self-concept. The group was underpowered. Both the assessments and interventions were delivered unblinded by the researcher and we calculated that there was no effect on the children’s motor ability.

### Limitations and future research

It was inevitable that some limitations remain in the included studies (e.g. failure to blind participants, unmasked outcome assessors) despite this review excluding studies of low methodological quality. A score of seven out of 11 on the PEDro scale still indicates that four important features indicative of high methodological quality were omitted, suggesting the potential for bias. While we recognize that not all criteria within the PEDro checklist can reasonably be met in rehabilitation trials (e.g. blinding of participants), blinding of assessors is essential for more robust results. True randomization is essential for reducing bias, but participants were often only quasi-randomly allocated to groups (e.g. by school). Other limitations were not including a non-treatment group, or not describing the comorbidity or heterogeneity of the children.

It is disappointing that some evidence is weak because of underpowered trials. A number of studies lacked power calculations or used small sample sizes (*n* < 20), a methodological flaw not assessed by the PEDro Scale. At least two studies in this review had a probability of less than 50% of detecting a true effect. Thus, we strongly recommend that investigators perform a priori statistical power calculations and include the outcome of this procedure in their articles. We also recommend greater collaboration between investigators to prevent Type-II errors through high-powered studies.

One further possible limitation within this review is the unassessed but likely influence of publication bias.^27^ Despite the intense interest in this area over the last two decades, it would appear that only a few studies of high methodological quality have been undertaken. Knowing the bias against publication of negative findings,^26^ it is conceivable that equally high quality but unpublished studies with negative conclusions remain unreported. In light of this possibility, we would strongly emphasize the need for mandatory preregistration of all future randomized controlled trials of motor interventions in developmental coordination disorder.^96^

We also recognize that while the methodological approach taken within this article (a systematic review of randomized controlled trials) is widely considered to be one of the best methods for synthesizing knowledge in order to guide clinical decision making, it is not without its limitations. Systematic reviews are useful for information, but not a substitute for a clinician’s analytical judgements^97^ and must be acknowledged as containing elements of subjectivity. For example, despite the PEDro scale having good inter-rater reliability,^98^ low-level variability did still lead to some disagreements between how this review and previous systematic reviews classified certain studies’ quality level.^11^ It should also be kept in mind that in particularly heterogeneous conditions (such as developmental coordination disorder) there is likely to be imperfect agreement between the clinical reality of how effective an intervention is for any given patient and the efficacy level reported in tightly controlled randomized controlled trials.^99^

## Conclusion

This systematic review investigated interventions for improving motor skills of children with developmental coordination disorder and found that task-orientated approaches such as Neuromotor Task Training, ‘conventional’ motor training interventions (such as those commonly used by occupational therapists and physiotherapists) and motor imagery training combined with practice of the motor tasks may yield positive benefits. However, in each respect only a single study has been judged as being of sufficient methodological quality to arrive at this conclusion and all reflect comparatively small sample sizes from single-centre studies. To date, no large-scale multisite randomized control trial (with adequately blinded assessors) has been conducted in relation to any motor skills intervention intended to remediate the impact of developmental coordination disorder. What is clearly evident is that certain features seem to be shared by all effective interventions (e.g. a task-orientated approach) and may be useful as guiding principles in future research into an effective intervention. In addition, the heterogeneity of children with developmental coordination disorder should be taken into account.

Wii, core stability training, self-concept training, Tai Kwon Do, table tennis and aquatic therapy are not supported by the available evidence and on the basis of the empirical evidence their usage is not recommended: Effect sizes for these interventions are negligible, small or there is stronger evidence for more effective interventions.

With respect to future research in this area, power calculations are necessary when designing a study and blinded assessors are essential for reducing biased outcomes. We recommend the inclusion of more complete descriptions of the participants and the use of pretrial registration to mitigate for publication bias.

**Clinical messages**

- Suggests the most effective interventions on the basis of randomized controlled trials with high methodological quality are task-oriented interventions.
- Suggests that Wii Fit and psychological (self-concept training) are ineffective.
- Highlights that evidential quality is commonly lowered by the absence of power calculations and blinded assessors.

## Supplementary Material

Supplementary material
