# Supplementary material for: A systematic review of high quality randomized controlled trials investigating motor skill programmes for children with developmental coordination disorder
Source: Clin Rehabil. 2016 Aug 1;31(7):857–70. doi: 10.1177/0269215516661014 (PMC5482383; doi:10.1177/0269215516661014)
Supplement: Supplementary material [file CRE661014_Online_Tables_CLN.doc]

Table 1. The PEDro scale.

|  | **Criteria** Each criterion scores 1 point: criterion 1 relates to generalizability of study; criteria 2 – 9 relate to internal validity; criteria 10 – 11 relate to statistical reporting sufficient to interpret results.^16^ |  | **Yes** |  | **No** |
| --- | --- | --- | --- | --- | --- |
|  | Eligibility criteria were specified |  |  |  |  |
|  | Subjects were randomly allocated to groups (in a crossover study, subjects  were randomly allocated an order in which treatments were received) |  |  |  |  |
|  | Allocation was concealed |  |  |  |  |
|  | The groups were similar at baseline regarding the most important prognostic indicators |  |  |  |  |
|  | There was blinding of all subjects |  |  |  |  |
|  | There was blinding of all therapists who administered the therapy |  |  |  |  |
|  | There was blinding of all assessors who measured at least one key outcome |  |  |  |  |
|  | Measures of at least one key outcome were obtained from more than 85%  of the subjects initially allocated to groups |  |  |  |  |
|  | All subjects for whom outcome measures were available received the Treatment or control condition as allocated or, where this was not the case, Data for at least one key outcome was analysed by “intention to treat” |  |  |  |  |
|  | The results of between-group statistical comparisons are reported for at least one key outcome |  |  |  |  |
|  | The study provides both point measures and measures of variability for at  least one key outcome |  |  |  |  |

Supplementary Table 1. Search terms for systematic review. Columns were combined using the OR facility; column results were combined using the AND facility. Wildcards (e.g. *, ?) widened the scope of search terms: see row 2 for an example.

|  | **P**opulation | **P**atients | **I**ntervention | **O**utcome |
| --- | --- | --- | --- | --- |
|  | children | developmental coordination disorder | cognitive orientation to occupational performance | fine motor |
|  | paediatrics  (p?ediatric*) | *praxia | CO-OP [Cognitive Orientation to Occupational Performance] | gross motor |
|  | infants | Deficit in attention motor control and perception | physiotherapy | balance |
|  | toddlers | DCD | physical therapy | posture |
|  | school children | clumsy | occupational therapy | handwriting |
|  |  | Nonverbal Learning Disorder | Tai Kwon Do | motor coordination |
|  |  | Awkward | table tennis | motor performance |
|  |  |  | sports | movement |
|  |  |  | sensory integration | occupational performance |
|  |  |  | SI | clumsiness |
|  |  |  | le bon depart | functional |
|  |  |  | core stability | activity |
|  |  |  | motor training | motor impairment |
|  |  |  | task oriented | movement |
|  |  |  | exercise training | motor ability |
|  |  |  | virtual reality | motor control |
|  |  |  | computer | motor function |
|  |  |  | metronome | motor learning difficulty |
|  |  |  | dynamic performance analysis | dysgraphia |
|  |  |  | motor skill training | motor development |
|  |  |  | treatment | motor dysfunction |
|  |  |  | psychomotor | motor skills |
|  |  |  | movement | motor learning |
|  |  |  | postural control | motor performance |
|  |  |  | balance |  |
|  |  |  | motor learning |  |
|  |  |  | physical education |  |
|  |  |  | PE |  |
|  |  |  | physical training |  |
|  |  |  | rehabilitation |  |
|  |  |  | intervention |  |

Supplemetary table 2. Description of the nine studies meeting criteria for high quality evidence.

| **Randomized controlled trials (N = 9)** | | | | | | | | | | | |
| --- | --- | --- | --- | --- | --- | --- | --- | --- | --- | --- | --- |
| **Author** | | **Aim/objectives of the study** | **Study design (PEDro score out of 11)** | **Study inclusion and exclusion criteria** | **Motor skills outcome measures** | **Adequate power?** | | **Recruitment procedures used (e.g. details of randomization, blinding)** | | **Description of the intervention(s) and control(s)** | |
| Wilson, Thomas & Maruff, 2002^93^ | | To investigate whether imagery training is equally effective to perceptual-motor training in developing the motor skills of children with coordination problems. | Single-blind randomized controlled trial (8/11). | Children aged 7-12 years with poor motor coordination. Exclusion criteria: current/past history of neurologic disease, including head injury, psychiatric disorders, or ADD. | MABC-1 | No power calculations included. Group sizes suggest that the study achieved adequate power, based on other study calculations. | | Blocked randomization procedure to balance the groups within four PC ranges of total impairment. All children assessed by blinded assessor. Three intervention groups: (1) imagery training, (2) traditional perceptual-motor training, and (3) wait-list control.  (n=18 in each group) | | **Imagery Training Group**: Individual intervention base on: PC-based program of (a) visual imagery exercises involving predictive timing, (b) relaxation protocol and mental preparation, (c) visual modelling of fundamental motor skills, (d) mental rehearsal of skills from an external perspective, (e) mental rehearsal of skills from an internal perspective, and (f) overt practice. **Traditional Perceptual-Motor Training Group**: Individual intervention constituted of a combination of fine and gross motor, and perceptual-motor activities tailored to the individual needs. Average time period between pre- and post-testing: 4.5 weeks ± 4 days. | |
| S. S. M. Fong, W. W. N. Tsang and G. Y. F. Ng  2012^87^ | | To investigate the effect of 3 months intensive TKD training on the sensory organization and balance performance of children with DCD | Single-blind stratified randomized controlled trial  (7/11) | Inclusion criteria:  (1) DCD according to the DSM-IV-TR; (2) 6-9 years; (3) study in a regular education framework; and, (4) no intellectual impairment.  Exclusion criteria: (1) receiving physical or occupational therapy training; or (2) demonstrated excessive disruptive behaviour; or (3) could not follow instructions thoroughly. TDC (n=18): same criteria  Except not having DCD. | Sensory organization of balance control: somato-sensory ratio, visual ratio, vestibular ratio. Single leg standing balance: unilateral stance centre of pressure sway velocity. | Power calculated at 19 participants per group; study achieved adequate power in all groups except the TD control. | | Testers blinded to allocation of participants. Participants stratified by gender and then randomly assigned to either the DCD-TKD training group (n=21) or the DCD-CG (n=23) by drawing lots and was completed by a person independent of the study. | | **DCD-TKD training group**: weekly group intervention 1-h session of TKD training for 12 consecutive weeks. Protocol modified from a typical TKD syllabus for beginners. TKD training sessions conducted by a World Tai Kwon Do Federation 4th Dan black belt and a 2nd Dan black belt. Similar TKD home exercises (one hour in duration) performed daily throughout the 3 month study period. Guardians provided with written instructions and asked to coach/assist their children in performing the TKD home exercises. The **DCD-control and normal-control groups** received no training within the study period. | |
| G. D. Ferguson, D. Jelsma, J. Jelsma and B. C. M. Smits-Engelsman  2013^89^ | | To compare the efficacy of NTT and Nintendo Wii Fit training in two groups of children with DCD attending schools in a low socioeconomic area. | Quasi-randomized controlled study  (8/11) | Children aged 6 - 10. Motor performance below the 16th PC on the MABC and presence of a motor coordination problem interfering with activities in daily life. Exclusion criteria: repetition of any grade level more than once and diagnosis of significant medical disorder. | MABC-2 | Power calculated at 17 participants per group; study achieved adequate power (27 and 19 children per group). | | Schools A & B children received NTT (n = 27) and School C children received Nintendo Wii training (n = 19). Quasi-randomized design due to power problems at schools A and B prohibiting use of Wii. Apart from the functioning power supply, there were no significant differences between schools in terms of playground facilities, socioeconomic backgrounds of the learners, school fees, staff ratios or curriculum. | | **Wii group**: 30 mins of Nintendo Wii Fit balance board, 3 times a week for 6 weeks (group intervention). Training sessions included 13 games involving mimic the act of cycling, soccer, skateboarding and skiing. Additionally, 5 games incorporating arm movements using the hand held controller were also used. **NTT program**: 9 weeks, 2 sessions/week each lasting 45-60 min (group intervention). Two therapists identified the main motor control problems. Workstations were set up where children could practice components of soccer, netball, variations of tagging games and other popular games under the guidance of the therapists who manipulated aspects of the environment and task as needed. | |
| S. Hillier, A. McIntyre and L. Plummer  2010^90^ | | To determine whether aquatic therapy is feasible for children with DCD, and provide preliminary data regarding the effectiveness of aquatic therapy in improving the motor skills, self-concept, and participation of children with DCD. | Single-blinded randomized controlled trial (9/11). | 5-8 years of age. Inclusion and exclusion criteria: criteria for DCD diagnosis in DSM-IV. Also exclusion if currently/recently attended swimming lessons; frequently using a pool; comorbidities, including intellectual disabilities; had hydrophobia or other hydrotherapy contraindications. | MABC-1 | Power calculated at 19 participants per group for 90% power; study failed to achieve adequate power (six children per group). | | Independent random allocator using a computer program. | | Aquatic therapy: six, 30-min aquatic physiotherapy sessions in a 1 to 1 format. The 6 sessions were scheduled over a 6–8-week period, aiming for one per week. The wait-list CG was advised to continue as usual. | |
| C. L. Tsai, C. H. Wang and Y. T. Tseng  2012^94^ | | To explore whether soccer training would have a beneficial effect on the performance of a visuospatial attention task and improve the inhibitory control capability in children with DCD. | Quasi randomized controlled trial (9/11). | 9-10 years old. DCD according to DSM-IV-TR, with the total impairment score below the 5th PC cut-off point | MABC-1 | No power calculations included. Group sizes would suggest study lacks power compared to other studies. | | Quasi-random allocation to intervention (n=16) and CG (n=14). Total motor impairment and age distributions were similar between the two groups before exercise training. | | DCD-training group: group intervention of 50-minute soccer training sessions, 5 times/week, ten weeks. DCD non-training group and TDC group performed their regular classroom activities and did not participate in any training. The coach was a special education and physical education teacher blinded to the children’s characteristics of motor impairment. | |
| Hung & Pang, 2010^91^ | | To compare the effects of group- and individual-based motor skill training on motor performance in children with DCD | Randomised controlled. pilot trial (8/11) | 6-10 years with a diagnosis of DCD. Gross motor composite score of < 42 as measured by the BOTMP. Children excluded if: (1) had received or were undergoing physical therapy or occupational therapy; (2) had any visual or hearing deficiencies that could not be corrected by external devices; (3) demonstrated excessive disruptive behaviour; and (4) total impairment score of greater than the 15th PC on the MABC | MABC-1 | No power calculations included. Group sizes would suggest study lacks power compared to other studies. | | Participants were randomly allocated to either individual- (n=11) or group- (n=12) intervention. The randomization procedure was performed by a researcher not involved in the assessment of the children. | | The subjects underwent a group intervention weekly 45-min session of motor skill training for 8 consecutive weeks. The activities involved in the individual and group training were essentially the same. Each subject was given home exercises. | |
| Peens, Pienaar & Nienaber 2008^92^ | | To examine whether a pure motor-based, an integrated psycho-motor or a psychologically based intervention will be the most effective in enhancing the motor proficiency of children with DCD. | randomised controlled trial (7/11). | 7-9 year old children with DCD (MABC used for the allocation) | MABC-1 | Power calculated at 12 participants per group; only motor based group and control group achieved adequate power. | | Four intervention groups: (1) Motor-based (n=20), (2) Psychological (n=10), (3) Integrated psycho-motor (n=11) and (4) CG (n=17). Randomization methods not explained. Neither intervention supervisor nor assessments were blinded: the researcher conducted both testing of the children and the intervention program. | | **Psychological (self-concept enhancing) intervention program**; Group and individual intervention,1 x 45 min per week for eight weeks; centred around discovering of the self, self-acceptance, enhancing self-concept. Included one session for parents on parenting skills. **Motor-based intervention program**: group intervention, 2 x 30 min per week for 8 weeks; introduction of locomotor activities, Ball Skills (2–3 act.), Balance Skills (2–3 act.), fine motor co-ordination (2 act.) and eye control (1 act.). The primary researcher conducted the program (progressively adapted, once a week). **Psycho-motor intervention program**: group intervention, each week, 2 x 30 min motor-based intervention sessions + one 45 min psychological intervention session per week for 8 weeks. | |
| Tsai 2009^95^ | | To explore the effectiveness of table tennis training on inhibitory control of DCD; and to explore the efficacy of this intervention on motor skills. | Non-randomized single-blind controlled trial (8/11) | Twenty seven 9-10 years old. DCD group inclusion & TDC group exclusion criteria: total impairment score at or below the 5th PC cut-off point of the MABC. Children with any definite signs of special educational needs, physical or behavioural problems, or evident neurological damage were also excluded. | MABC-1 | No power calculations included. Group sizes would suggest the study lacks power compared to other studies (14 in each group). | | Quasi-random allocation of 28 participants to DCD-training group or a DCD non-training group. TDC were randomly selected from the normal sample. The coach was blind to the participants’ characteristics of motor impairment. | | Table tennis training program was aimed at improving general skills. 50-min sessions of group intervention, 3 times/week for ten weeks. Each skill began with a simple movement and then progressed to more complex variations. After 2 weeks of training, more task-specific training was added into the main program. | |
| M. K. Au, W. M. Chan, L. Lee, T. M. Chen, R. M. Chau and M. Y. Pang  2014^88^ | | To compare the effectiveness of core stability program (based on the process-oriented approach) and task-oriented motor program in improving motor proficiency of children with DCD. | randomized controlled pilot trial (9/11) | Children (6-12 years) met criteria for DCD in DSM-IV and scored lower than 15th PC on the MABC. Excluded if: had attended treatment for motor problems in the previous six months, or had any major co-morbid medical problems. | BOTMP short form as the primary outcome. The Sensory Organization Test was conducted to evaluate the sensory organization of postural control. | | No power calculations included. Group sizes would suggest the study lacks power compared to other studies. | | Randomization conducted by a researcher who was not involved in selection, assessment and treatment. All assessments were conducted by a paediatric physiotherapist who was blinded to group assignment. | | Each program consisted of a one-hour training session/week for 8 weeks of group intervention. **Core stability training** (n=11): a physioball was used as the treatment tool. **Task-oriented training** (n=11): training functional tasks, including mainly body stability and body transport of group intervention. Children in each group were also asked to perform the exercises daily at home, as taught in the face-to-face sessions. Home exercise sheets were given to the parents. An exercise log book was provided to each child, so that their compliance with the home program could be recorded. |
|  | Abbreviations:  PC: percentile; ADD: Attention Deficit Disorder; TDC: Typically Developing Children; CG: control group; TKD (Tai Kwon Do; DCD: Developmental Coordination Disorder; randomised controlled trial: Randomized Controlled Trial; SD: standard deviation; NTT: Neuromotor Task Training; MABC: Movement Assessment Battery for Children; BOTMP: Bruininks-Oseretrsky Test of Motor Proficiency; CT: Clinical Trials. | | | | | | | | | | |

Supplementary table 3. Results of each study and Summary of review conclusion.

| **Randomized controlled trials (n = 9)** | | | |
| --- | --- | --- | --- |
| **Author** | **Statistical techniques used** | **Results** | **Summary** |
| Wilson, Thomas & Maruff, 2002^93^ | Two-way repeated measures analysis of variance. To complement the use of significance testing, estimates of effect size (eta-squared, η^2^) were provided for group comparisons and interpreted according to the statistical conventions of Cohen. Pearson’s product-moment correlations were calculated between pretest scores on the MABC and change scores for each group. | Results of outcome measures were not given in detail but were described as follows:  Average change scores: 4.15 (SE = 0.91) for the imagery training group (F(1,48) = 15.90, P < 0.001, η^2^ = 0.30); 5.09 (SE = 1.31) for the perceptual motor-training group, (F(1,48) = 23.93, P < .001, η^2^ = 0.33); and 1.38 (SE = 0.83) for the CG, (F(1,48) = 1.77, P = 0.190, η^2^ = 0.04).  Calculated effect sizes: imagery training group d = 4.8, 95% CI: 3.51 to 6.09  perceptual motor-training group d = 4.6, 95% CI: 3.35 to 5.85    There was no effect in the CG and no difference between imagery training groups. | The imagery protocol was equally effective to perceptual-motor training in facilitating the development of motor skill in the children with movement difficulties Both showed a large effect size. No changes were seen in the CG. No power calculations were given for this study. |
| S. S. M. Fong, W. W. N. Tsang and G. Y. F. Ng  2012^87^ | Two-way repeated measures multivariate analysis of covariance (MANCOVA) was conducted incorporating all the outcome measures. The within-subject factor was time and the between-subject factor was group. The intention-to-treat principle was employed. If the MANCOVA demonstrated a significant effect overall, follow-up analyses were performed using one-way ANCOVA and post-hoc pairwise comparisons. | The average vestibular ratio of the DCD-TKD group was 61.8% higher than that of the DCD-CG and comparable to that of the normal-control group (p > 0.01). DCD-TKD group demonstrated the greatest improvement over time (18.5%, p = 0.001), followed by the DCD-CG (5.8%, p = 0.023). Within-group differences were not significant (p > 0.05) in the normal-CG. However, there was no difference (p > 0.01) in the composite scores among the three groups pre-test or post-test. DCD-TKD children swayed 30.5% slower when standing on one leg after TKD training (p = 0.004), comparable to that of their TD peers (p > 0.05). The DCD-CG (without TKD training) did not improve over time (p > 0.05) and their post-test UST COP sway velocity was 121.6% higher than that of the normal-CG (p = 0.001) and 71.5% higher than that of the DCD-TKD (p = 0.007) group.  Calculated effect sizes: general motor control composite score d = 0.80, 95% CI:0.19 to 1.41; and COP sway velocity d = 0.48, 95% CI: -0.12 to 1.08. | Confidence intervals suggest that TKD training potentially had no effect on balance. There is no indication that there were any functional mobility improvements. |
| G. D. Ferguson, D. Jelsma, J. Jelsma and B. C. M. Smits-Engelsman  2013^89^ | The GLM was used (time of assessment (i.e., pre- and post-training) as the within-subjects factor and treatment group as the between-subjects factor). If appropriate, t-tests were used to determine how groups differed between the two assessments. Effect sizes (d) were calculated to determine the practical significance of these differences. d-values of 0.5 were taken to indicate a moderate effect and values greater than 0.8 were taken to indicate a large practical significance. | The ANOVA suggested a significant difference across time (within groups) (F(1,44) = 33.27, p < 0.001, η^2^ = 0.43) and a significant interaction between time and group (F(1,44) = 33.27, p < 0.001, η^2^ = 0.43). Results taken from Table 1, Ferguson et al^23^  t-tests were then conducted:  NTT group: mean standard MABC score change = 4.41 (SD 2.7), t(26) = -9.92, p < 0.01, d = 3.52, 95% CI: 2.59 to 4.45  Wii group: mean standard MABC score change = 0.73 (SD 3.17), t(18) = -1.16, p= 0.26, d = 0.58, 95% CI: -0.02 to 1.18. | NTT showed a statistically significant and very large change in MABC scores. Of note was that the group's manual dexterity, which was not practiced as part of this intervention, showed a statistically significant and large improvement. The Wii group confidence intervals suggest a potentially trivial effect on motor skills. |
| S. Hillier, A. McIntyre and L. Plummer  2010^90^ | MABC total impairment scores were analysed using ANCOVA. The subtest scores and total scores on the PSPCSA were analysed by a non-parametric ANCOVA. The scores of each group on individual questions of the participation questionnaire were analysed by the Mann–Whitney U-test. An effect size for the primary outcome (MABC) was calculated as a standardized mean difference with 95% confidence intervals, in Revman5, using the post-test means and standard deviations, and participant numbers for each group. | There was no statistically-significant change in any outcome measure including the MABC (pre-test score = 19.4, post-test score = 15.54, mean change 3.9, F(1, 9) = 4.78, *p* = 0.057). This 20% improvement in scores represents a moderate effect size (d = 0.66, 95% CI: -0.50 to 1.82). | This study suggests that aquatic therapy is feasible if convenient for attendance, but the confidence intervals suggest potentially no effect at all. |
| C. L. Tsai, C. H. Wang and Y. T. Tseng  2012^94^ | Repeated measure ANOVA, with time of assessment (pre- and post-training) as the within-subjects factor and group as the between-subjects factor. To control for any differences that might have existed prior to the soccer training, post-training data always accounted for pre-training data using ANCOVA. The averaged pre-training measures were used as the covariate. Estimates of effect size (partial g2) were provided for group comparison partial g2 < 0.08 (small effect size), partial g2 between 0.08 and 0.14 (medium effect size), and partial g2 > 0.14 (large effect size), except for the comparison of ‘time of assessment,’ in which corrected eta-squares were presented. | Change in mean MABC scores:  DCD-training group improved by 5.5 (SD 5.8), a statistically significant improvement with a large effect size d = 1.23, 95% CI: 0.45 to 2.01, (t(15) = 5.63, p < 0.001).  DCD-non training group showed an improvement of 1.18 (SD 4.8), this was not statistically significant and the effect size was trivial d = 0.28, (t(13) = -2.03, p = 0.063).  The non-training TDC group also showed no change: t(20) = 0.38, p = 0.710]. | The results of this study suggest that soccer training had a potential effect ranging from small to large effect on the motor skills of children with DCD. |
| Hung & Pang, 2010^91^ | To assess the effect of each type of intervention on motor performance (the within-group effect), Wilcoxon-signed rank tests were used to compare the MABC total impairment score and the subtest scores before and after the intervention for each group. The Mann-Whitney U tests were then used to compare the change in total impairment score and subtest scores (the post-test score minus the pre-test score) between the 2 groups (the between-group effect). The Mann-Whitney U test was also used to compare the home exercise compliance rate and parental satisfaction scores of the 2 groups. | A significant reduction in the MABC total impairment score was found following both group-based (mean difference –4.4 (SD 5.0), p = 0.003, effect size d = 1.14, 95% CI: 0.26 to 2.02 ) and individual-based training (mean –5.2 (SD 5.1), p = 0.016, effect size d = 1.35, 95% CI: 0.44 to 2.26). This change was not different between the groups (p = 0.379). There was no significant between-group difference in home exercise compliance. | The study suggests that motor skills improve equally after group-based and individual-based motor skills training, and there is no difference between approaches. Both produce an effect size confidence interval ranging from small to large. The study conducted no power calculations, and group sizes were small. |
| Peens, Pienaar & Nienaber 2008^92^ | A two-way ANOVA was used to evaluate differences between groups and differences within groups over time. When an interaction was encountered, a repeated-measures ANOVA for each group was conducted followed by a Bonferroni post hoc analysis to determine how the different groups differ between the four testing periods (within group differences). A one-way ANOVA followed by a Tukey post hoc analysis was also conducted to determine between-group differences at the pre-, post- and retests. | Mean MABC change scores:  Motor intervention group scores improved from 16.28(5.54) to 6.53(5.87), mean change 9.75 (SD 8.1), p < 0.001, effect size d = 1.47, 95% CI: 0.74 to 2.20.  Psychological intervention targeting self-concept: 15.55(5.30) to 15.6 (8.26), mean change 0.05 (SD 9.8), p = 0.32, trivial effect size d = 0.01, 95% CI: -0.77 to 0.79.    Psycho-motor intervention improved from 17.73 (6.74) to 9.64(8.58), mean change 8.09 (SD 10.9), p < 0.001, large effect size = 1.13, 95% CI: 0.32 to 1.94.  The no intervention control group improved from 17.09 (7.54) to 10.82 (9.18), mean change 6.27 (11.9), p < 0.001, effect size d = 0.83, 95% CI: 0.25 to 1.42. | The study showed a statistically significant effect size for motor performance in the motor training group ranging from medium to very large. The combined psychological/motor group produced an effect size confidence interval ranging from small to large. The self-concept (psychological) group showed no effect based on its confidence interval.  The CG which underwent no intervention also showed an effect size which ranged from small to large, possibly suggesting that the results of this underpowered study are not representative of true effects. |
| Tsai 2009^95^ | A two-way repeated-measures ANOVA was used to evaluate changes in total MABC scores, with time of assessment as the within-subjects factor and group as the between-subjects factor. To control for any differences that might have existed prior to the table tennis training, post-training data always accounted for pre-test data using ANCOVA procedure. | The ANOVA suggested that there were statistically significant differences for  time [F(1,40) = 4.84, p = .034, partial h2 = 0.11], groups [F(2, 40) = 112.48, p < .001, partial h2 = 0.85] and time x group [F(2, 40) = 5.26, p =.009, partial h2 = 0.21].  Mean (SD) change in MABC scores:  DCD-training group showed a statistically significant improvement of 4.31 (5.1), t(12) = 4.02, p = .002, effect size = 0.95, 95% CI: 0.15 to 1.75.  DCD-non training group showed a statistically non-significant change of 1.1 (6.2) , t(13) = 0.78, p = 0.451, effect size = 0.2.  TD control group showed a statistically non-significant change of 0.97 (3.4), t(15) = -0.97, p = 0.345, effect size 0.49. | Ten weeks of table tennis training produced a confidence interval ranging from trivial to large. This suggests potentially no benefit of table tennis training on motor skills of children with DCD. |
| M. K. Au, W. M. Chan, L. Lee, T. M. Chen, R. M. Chau and M. Y. Pang  2014^88^ | Within-groups changes were compared using Wilcoxon Signed Ranks tests. Mann-Whitney U test was used to compare the change score (post-test score minus pre-test score) of motor proficiency and Sensory Organization Test-derived scores between the two groups.  A level of significance of 0.05 was set, except for the within-group comparison of the pre-test and post-test data, where a more stringent level of significance (P ≤0.025) was used to reduce the risk of making a type I error associated with multiple comparisons. | Mean changes in BOTMP scores:  The core stability group showed a mean improvement of 6.3 (CI 2.7 to 9.9), p <0.025, effect size d = 1.22, 95% CI: 0.31 to 2.13.  The task-oriented group showed a mean improvement of 5.1 (CI 2.4 to 7.8), p < 0.025, effect size d = 0.98, 0.10 to 1.86 ;  Effect size was larger in the core stability group but there was no statistically significant difference between groups (p = 0.717).  The composite equilibrium score was significantly improved after the 8-week training period in the task-oriented group (P = 0.009, moderate effect size d = 0.46), but not in the core stability group (mean change = 0, p = 0.812. More than 80% of the parents, regardless of the group assignment, preferred group-based training than individual-based training.  In the task-oriented group only, there was a strong correlation between children’s participation in the home exercise program and improvement in both the motor scores (ρ = 0.680, p = 0.03) and composite equilibrium scores (ρ = 0.638, p = 0.047). | The results suggested that both the core stability program and task-oriented motor program have similar effects on enhancing motor proficiency in children with DCD. Firm conclusions cannot be drawn, however, due to the small sample size which produced a between-groups power of only 0.47. |
| Abbreviations:  PC: percentile; ADD: Attention Deficit Disorder; TDC: Typically Developing Children; CG: control group; TKD (Tai Kwon Do; DCD: Developmental Coordination Disorder; randomised controlled trial: Randomized Controlled Trial; SD: standard deviation; NTT: Neuromotor Task Training; MABC: Movement Assessment Battery for Children; BOTMP: Bruininks-Oseretrsky Test of Motor Proficiency; GLM: General Linear Model; PSPCSA: Pictorial Scale of Perceived Competence and Social Acceptance; ANOVA: analysis of variance; ANCOVA: analysis of covariance; MANCOVA: multivariate analysis of covariance | | | |
